# DeepEP: a deep learning framework for identifying essential proteins

**DOI:** 10.1186/s12859-019-3076-y

**Published:** 2019-12-02

**Authors:** Min Zeng, Min Li, Fang-Xiang Wu, Yaohang Li, Yi Pan

**Affiliations:** 1 School of Computer Science and Engineering, Central South University, Changsha, 410083 People’s Republic of China; 2 Division of Biomedical Engineering and Department of Mechanical Engineering, University of Saskatchewan, Saskatoon, SKS7N5A9 Canada; 3 Department of Computer Science, Old Dominion University, Norfolk, VA23529 USA; 4 Department of Computer Science, Georgia State University, Atlanta, GA30302 USA

**Keywords:** Deep learning, Identifying essential proteins, node2vec, Imbalanced learning, Protein-protein interaction network, Multi-scale convolutional neural networks

## Abstract

**Background:**

Essential proteins are crucial for cellular life and thus, identification of essential proteins is an important topic and a challenging problem for researchers. Recently lots of computational approaches have been proposed to handle this problem. However, traditional centrality methods cannot fully represent the topological features of biological networks. In addition, identifying essential proteins is an imbalanced learning problem; but few current shallow machine learning-based methods are designed to handle the imbalanced characteristics.

**Results:**

We develop DeepEP based on a deep learning framework that uses the node2vec technique, multi-scale convolutional neural networks and a sampling technique to identify essential proteins. In DeepEP, the node2vec technique is applied to automatically learn topological and semantic features for each protein in protein-protein interaction (PPI) network. Gene expression profiles are treated as images and multi-scale convolutional neural networks are applied to extract their patterns. In addition, DeepEP uses a sampling method to alleviate the imbalanced characteristics. The sampling method samples the same number of the majority and minority samples in a training epoch, which is not biased to any class in training process. The experimental results show that DeepEP outperforms traditional centrality methods. Moreover, DeepEP is better than shallow machine learning-based methods. Detailed analyses show that the dense vectors which are generated by node2vec technique contribute a lot to the improved performance. It is clear that the node2vec technique effectively captures the topological and semantic properties of PPI network. The sampling method also improves the performance of identifying essential proteins.

**Conclusion:**

We demonstrate that DeepEP improves the prediction performance by integrating multiple deep learning techniques and a sampling method. DeepEP is more effective than existing methods.

## Background

Essential proteins are indispensable for organisms and play a very important role in maintaining cellular life [1, 2]. Determination of essential proteins not only helps us understand the basic requirements of a cell at a molecular level, but also helps identifying essential genes and finding potential drug targets. Thus identifying essential proteins is very important for researchers. There are several biological experimental methods to identify essential proteins, such as RNA interference [3], conditional knockout [4], and single gene knockout [5]. But these methods require lots of resources and time. Moreover, in some complex organisms, these methods are not always applicable. Considering these experimental constraints, it is appealing to develop an accurate and effective computational approach for identifying essential proteins.

Existing computational approaches can be roughly divided into two categories: centrality methods and shallow machine learning-based methods. Jeong et al. [6] proposed centrality-lethality rule which point out that the highly connected proteins in a PPI network tend to be essential. Based on this rule, a lot of centrality methods have been proposed [7–12]. Meanwhile, researchers began to integrate more different useful biological information to identify essential proteins. A lot of different types of biological information, such as gene expression profiles [13, 14], subcellular localization information [15, 16], protein domains [17], orthologous information [18, 19], GO annotation and RNA-Seq data [20], have been used in various studies.

With the rapid development of high-throughput sequencing technique, we can easily get a lot of biological data which provide a solid foundation of using machine learning methods [21]. Generally, researchers develop a machine learning method for prediction according to the following steps: select some useful features (in this case, topological features of a PPI network), construct training and testing datasets, select an appropriate machine learning algorithm, and evaluate the performance of the algorithm. A number of shallow machine learning-based methods including support vector machine (SVM) [22], ensemble learning-based model [23], Naïve Bayes [24], decision tree [25] and genetic algorithm [26], are wildly used in identification of essential proteins.

Both centrality methods and shallow machine learning-based methods perform well, but each has some limitations. For centrality methods, current methods predict essential proteins by using a function to characterize the topological features of PPI networks according to their prior domain knowledge. But when the PPI network is very complicated (such as thousands of proteins and tens of thousands of protein-protein interactions), the function cannot characterize the topological features of such a complicated PPI network due to the output of the function is just a scalar [27, 28]. For shallow machine learning-based methods, the first step is selecting features. They usually select features by manual feature selection, which may pose a theoretical limitation to explain why these topological features are chosen in this study and depend heavily on the prior knowledge of researchers. In addition, identifying essential proteins is an imbalanced learning problem due to the number of non-essential proteins is much larger than the number of essential proteins. Data imbalance usually hinders the performance of machine learning methods, but few current shallow machine learning-based methods are designed to handle the imbalanced learning in essential proteins prediction.

To tackle the above limitations and further improve machine learning methods for identifying essential proteins, we propose DeepEP, a deep learning framework for identifying essential proteins. Recently, deep learning methods have been applied to represent network information and learn network topological features. They achieve the state-of-the-art performance in lots of applications [29, 30]. Inspired by their success, we aim to investigate whether deep learning methods could achieve notable improvements in the field of identifying essential proteins as well. We believe that deep learning techniques can be used to obtain better representation and thus improve performance. In particular, we employ the node2vec technique to encode a PPI network into a low-dimensional space, and then learn a low-dimensional dense vector for each protein in the PPI network. The low-dimensional dense vector represents the topological features of the corresponding protein. Using the node2vec technique has two advantages: (i) it provides a vector representation for a protein, this vector has a richer representation for topological features of a PPI network than a scalar; (ii) the node2vec technique can automatically learn vector representations from a PPI network and thus not require to choose some topological features. In addition, we use a sampling method to alleviate the imbalanced learning problem. The sampling method samples the same number of the negative samples (non-essential proteins) and positive samples (essential proteins) in a training epoch, and thus ensures the results are not biased to any class in training process. We use this strategy in many training epochs and can make full use of all non-essential proteins to train DeepEP with a high probability. In addition to overcoming the above limitations, DeepEP also uses other deep learning techniques to improve prediction performance. In this study, we use a PPI network dataset and gene expression profiles for training. For gene expression profiles, we transform them to images and thus we can use some deep learning techniques to better extract their patterns. Multi-scale convolutional neural network (CNN) is a newly developed deep learning architecture and is powerful for pattern extraction. We utilize it to extract more effective patterns of gene expression profiles.

To demonstrate the effectiveness of DeepEP, we perform extensive experiments on *S. cerevisiae* dataset. The experimental results show that DeepEP achieves better performance than traditional centrality methods and outperforms the shallow machine learning-based methods. To discover the vital element of DeepEP, we compare the results obtained by node2vec technique with those of 6 central methods. Detailed ablation study shows that the dense vectors which are generated by node2vec technique contribute a lot to the improved performance. Additionally, the sampling method also helps to improve the performance of identifying essential proteins.

## Materials and methods

### Overview: DeepEP

**Fig. 1:**
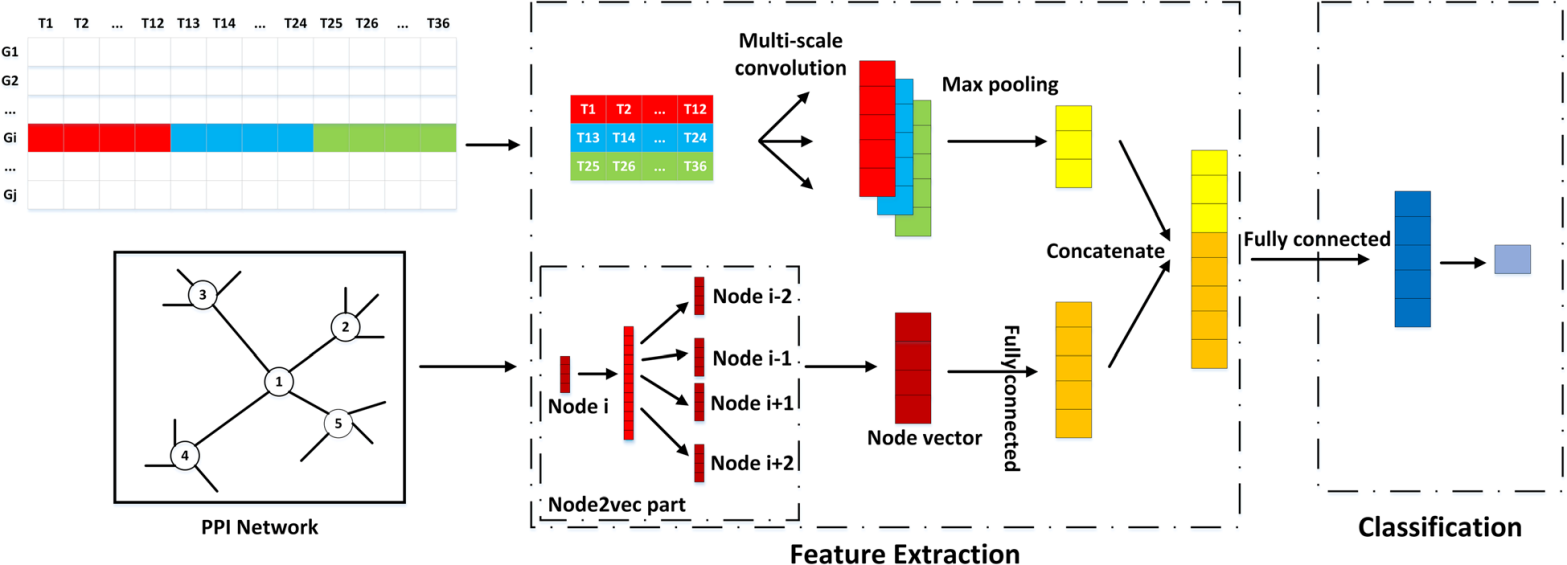
The architecture of our deep learning framework for identifying essential proteins

We propose a novel deep learning framework, DeepEP, for identifying essential proteins. Figure 1 illustrates the architecture of DeepEP. It consists of two major modules: a feature extraction module and a classification module. DeepEP accepts two kinds of biological datasets (PPI network dataset and gene expression profiles) as inputs. In the feature extraction module, the node2vec technique is applied to automatically learn a dense vector for each protein in a PPI network to capture the semantic and topological features of the biological network. Gene expression profiles are treated as images, and thus multi-scale CNN is applied to extract patterns. After multi-scale convolution layer, the pooling layer is used to perform dimension reduction. Then, the outputs of each component (node2vec technique, multi-scale CNN and pooling layer) are concatenated together as the inputs for classification module. The classification module consists of a fully connected layer and an output layer. A rectified linear unit (ReLU) function is applied to the fully connected layer as the activation function. After the fully connected layer, another fully connected layer with softmax activation function as output layer predicts the final label of a protein. In addition to using deep learning techniques, we also use a sampling method to alleviate the imbalanced learning problem. The details of the sampling method will be discussed in sampling method section.

### Network representation learning

As mentioned in the previous section, researchers need to select some useful features to accomplish the development of machine learning approach. Selecting PPI topological features is a very critical step in the study. Over the past 10 years, researchers proposed many effective computational methods to predict essential proteins based on network topological features such as DC, BC, CC, EC and so on. However, it is still difficult to select some centrality indexes from them. Traditional feature selection method used in identifying essential proteins is manual feature selection. There are two disadvantages in manual feature selection. The first one is that we have to must lots of prior knowledge about essential proteins. The second one is the selected topological feature is a scalar which cannot represent the complex topological features of a PPI network. To address the two issues, we use network representation learning technique to obtain biological features from a PPI network. Different from manual feature selection, network representation learning can automatically learn a low-dimensional dense vector for each protein in the biological network to represent the semantic and topological features. By using this technique, a dense vector which has more powerful representation than a scalar can be obtained and thus, it can improve the performance [31].

Various network representation learning techniques have been proposed in recent years [32]. Specifically, we used the node2vec technique [33] which can learn dense vector representations of vertexes in network based on deep learning methods. It uses biased random walk algorithm to generate a corpus which consists of every vertex’s sequence for training, and aims to predict the context of the given center node by maximizing the co-occurrence likelihood function. The node2vec technique can explore different types of networks and obtain richer topological representation of the network than traditional methods.

### Sampling method

Data imbalance is a very common phenomenon in real-world and we must take it into consideration in machine learning field. The imbalance problem is encountered in prediction of essential proteins. The classes that have more data instances are defined as the majority class, while the ones with fewer instances are the minority class. In the essential proteins dataset we used, the essential proteins belong to the minority class and non-essential proteins belong to the majority class. The imbalanced nature of data poses a challenge for identifying essential proteins. Most traditional machine learning methods usually bias towards the majority class and hence lead to loss of predictive performance for the minority class. Here our focus is to identify the essential proteins out of many non-essential ones, which requires us to tackle the problem of data imbalance effectively.

Previous studies have made great efforts to alleviate the imbalanced data learning problem. Sampling methods are the most wildly used and very effective methods [34–36]. However, we cannot direct use traditional sampling methods (random oversampling and SMOTE) in DeepEP due to the high consumption of computer resources. The vector which is fed to the classification module is a high-dimensional vector, and we do not want to synthesize any new samples for training based on the raw high-dimensional vector.

**Fig. 2:**
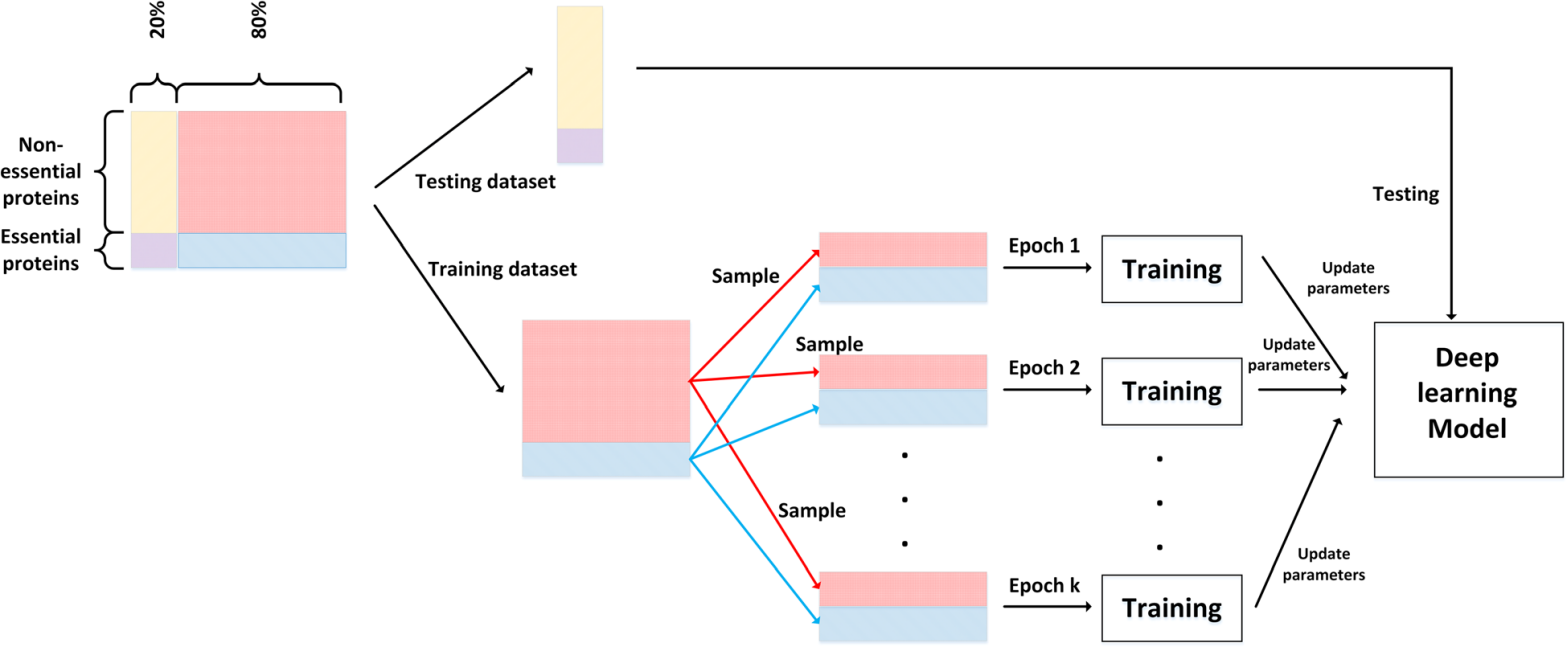
Illustration of the used sampling method

To alleviate the imbalanced learning problem, we use a low-computational cost sampling method. M and N denote the number of minority class samples (essential proteins) and the number of majority class samples (non-essential proteins), respectively. In each epoch, we sample M instances from the majority class, and then combine the M instances in the majority class and all instances in the minority class as a new subset to train DeepEP. We carry out this process k times to train DeepEP. The main advantage of using this sampling method is that it can ensure the results are not biased to any class in training process. Figure 2 gives the illustration of the sampling method.

In addition to the above advantage, the sampling method can make full use of all instances in the majority class of the raw dataset to train the deep learning model. In the above sampling process, at each epoch, the probability that a non-essential protein instance is picked is M/N. Therefore, for a specific non-essential protein, the probability that a non-essential protein is not picked at least once after k draws is:

1 $$ \mathrm{p}={\left(1-\mathrm{M}/\mathrm{N}\right)}^{\mathrm{k}} $$

In order to make this probability as small as possible, we can specify a threshold α to control it. If α is as small as possible, we believe that we have sampled all majority class instances of the raw dataset.

2 $$ {\left(1-\mathrm{M}/\mathrm{N}\right)}^{\mathrm{k}}<\upalpha $$

In this study, we set α =0.001, the training times k can be determined by Eq. (2).

### Multi-scale architecture

In order to better capture the patterns of gene expression profiles, we treat them as images. A gene expression profile has three successive metabolic cycles and each cycle has 12 time points. It is natural to regard one gene expression profile as an image with 1 channel * 3 rows * 12 columns, and thus some related techniques in computer vision can be applied in feature extraction for essential proteins prediction. Deep learning techniques have been successfully applied in computer vision and CNN is the most wildly used network architecture. CNN uses convolutional filters to extract local features [37] from raw images and multi-scale CNN uses different kernels to extract local contextual features [38]. By using different kernels, we obtain different information of different spatial scales. The combination of the information from the different scales can help to improve the prediction task. Figure 1 shows the illustration of how a gene expression profile is treated as an image.

### Assessment metrics

In order to evaluate the performance of DeepEP and other methods, in this study, we used six measures: accuracy, precision, recall, F-measure, area under the curve (AUC), and average precision (AP) score. Accuracy, precision, recall and F-measure are the most frequently used metrics in machine learning classification, they are defined as:

3 $$ Accuracy=\left( TP+ TN\right)/\left( TP+ TN+ FP+ FN\right) $$

4 $$ precision= TP/\left( TP+ FP\right) $$

5 $$ recall= TP/\left( TP+ FN\right) $$

6 $$ F- measure=\frac{2\ast precision\ast recall}{precision+ recall} $$

AUC is defined as the area under the Receiver Operating Characteristic (ROC) curve and ROC curve is a commonly used tool of visualizing performance of a classifier. AP score is defined as the area under the precision-recall (PR) curve and this assessment metric is widely used for evaluating identification of essential proteins. Note that F-measure, AUC, and AP score are more important than accuracy, precision and recall in an imbalanced learning problem due to they can offer a comprehensive assessment of a machine learning classifier.

### Datasets

We use three kinds of biological datasets in our experiments: PPI network dataset, essential proteins dataset, and gene expression profiles. The PPI network dataset is collected from BioGRID database [39]. To eliminate the noise of the dataset, we removed self-interactions and repeated interactions. There are 5616 proteins and 52,833 protein-protein interactions in the preprocessed PPI network dataset. The essential proteins dataset is collected from the four databases: MIPS [40], SGD [41], DEG [42], and SGDP. We removed some overlap proteins and integrated the information of the four databases. The preprocessed dataset of essential proteins contains 1199 essential proteins. The gene expression profiles dataset is collected from GEO database (accession number: GSE3431). It consists of 6776 gene products (proteins) and 36 samples. There are three successive metabolic cycles and each cycle has 12 time points.

## Results and discussion

### Implementation details

In our experiments, we first employ the node2vec technique to generate network representation vectors. Each protein in PPI network is represented by a 64-dimensional vector. Our deep learning framework is implemented by Tensorflow which is a wildly used deep learning system [43, 44]. Multi-scale CNN layers with kernel size 1, 3, and 5 are utilized to extract contextual features of gene expression profiles. By using multi-scale CNN layer we obtain 3 feature maps, each having 8 channels. These feature maps are concatenated together as the extracted contextual feature vector. Then the output of multi-scale CNN layer is fed to the maxpooling layer. After maxpooling layer, the output vectors and network representation vectors generated by node2vec are concatenated, and then the concatenated vector is fed to a fully connected layer which has 312 nodes with ReLU activation function. To avoid overfitting, a dropout rate of 0.1 is applied in DeepEP on fully connected layer. Finally, we train our deep learning framework using the Adam optimizer. The batch size is set to 32 and initial learning rate is set to 0.001.

### Comparison with other centrality methods

**Fig. 3:**
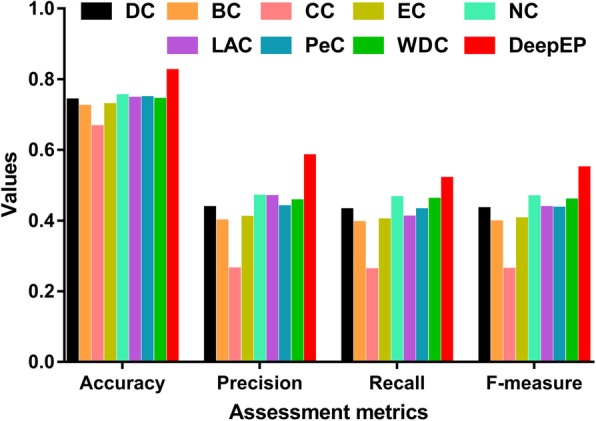
Performance of DeepEP, DC, BC, CC, EC, NC, LAC, PeC, and WDC

To demonstrate the effectiveness of DeepEP, we compared it with several popular centrality methods for essential proteins prediction. Eight centrality methods are used for the comparison. These centrality methods are used in following way. First, we compute the values of proteins in PPI network using each centrality method. Second, we rank their scores in descending order. Third, the top 1185 proteins are selected as candidate essential proteins. Last, we calculate precision, recall, F-measure and accuracy according to the true labels of proteins. The results of predicting essential proteins for each compared methods are shown in Fig. 3. As shown in Fig. 3, the results of DeepEP outperform the other centrality methods. For instance, the F-measure of DeepEP achieves the highest value. Similarity, other assessment metrics of DeepEP significantly are higher than those of other centrality methods. These results demonstrate the effectiveness of DeepEP for identifying essential proteins.

### Comparison with shallow machine learning-based methods

Machine learning-based methods are widely used in predicting essential proteins. SVM and ensemble learning-based model are the two most commonly used shallow machine learning-based methods. Besides, decision tree and Naïve Bayes are very popular methods. Thus these shallow machine learning methods (SVM, ensemble learning-based model, decision tree, Naïve Bayes) are compared to DeepEP. All of these shallow machine learning methods are implemented by scikit-learn python library with default parameters. We shuffle all samples in raw dataset and then split raw dataset into training dataset and testing dataset. Training dataset is composed of 80% samples of raw dataset and the rest samples constitute testing dataset. In both the training and the testing datasets, the ratio of positive samples (essential proteins) and negative samples (non-essential proteins) remains the same. We use two different ways to compare the machine learning-based methods. First, we directly utilize raw training dataset for training and testing on testing dataset. Second, we first apply the random undersampling technique to draw M (number of essential protein samples) samples from non-essential protein set of training dataset. Then we combine the selected non-essential proteins and all essential proteins together as input data to train machine learning models. The overall performance of all machine learning and deep learning algorithms are evaluated using testing dataset. To ensure a fair comparison, the input features are the same.

Table 1 gives a comparison of the experimental results of DeepEP with other shallow machine learning-based methods using different ratios for training. As shown in Table 1, we can see that the imbalanced nature of dataset hampers the mining of machine learning methods. F-measure and AUC increase from 0.21 and 0.72 (raw dataset) to 0.23 and 0.75 (1:1) by using random undersampling technique for SVM, from 0.35 and 0.58 (raw dataset) to 0.50 and 0.69 (1:1) for decision tree, from 0.27 and 0.70 (raw dataset) to 0.43 and 0.78 (1:1) for random forest, from 0.42 and 0.73 (raw dataset) to 0.43 and 0.75 (1:1) for Adaboost, and from 0.42 and 0.70 (raw dataset) to 0.44 and 0.71 (1:1) for Naïve Bayes. Other metrics of accuracy, precision and recall obtained in this work are also improved by using random undersampling technique except for the accuracy and precision of Adaboost (raw dataset). Our results show that it is necessary to consider the imbalanced nature of dataset. In addition, from Table 1, we conclude that DeepEP outperforms other machine learning-based methods. For instance, the F-measure and AUC of DeepEP are 0.55 and 0.82, respectively. They are higher than those of SVM (best performance: 0.23 and 0.75), decision tree (best performance: 0.50 and 0.69), random forest (best performance: 0.43 and 0.78), Adaboost (best performance: 0.43 and 0.75) and Naïve Bayes (best performance: 0.44 and 0.71).

**Table 1:** Performance of DeepEP and other shallow machine learning–based methods with different ratios

Machine learning algorithms	Accuracy	Precision	Recall	F-measure	AUC
SVM (raw dataset)	0.809	0.71	0.12	0.21	0.72
SVM (1:1)	0.813	0.75	0.14	0.23	0.75
Decision tree (raw dataset)	0.698	0.31	0.39	0.35	0.58
Decision tree (1:1)	0.781	0.47	0.54	0.50	0.69
Random forest (raw dataset)	0.809	0.63	0.17	0.27	0.70
Random forest (1:1)	0.843	0.74	0.31	0.43	0.78
Adaboost (raw dataset)	0.805	0.54	0.34	0.42	0.73
Adaboost (1:1)	0.785	0.47	0.39	0.43	0.75
Naïve Bayes (raw dataset)	0.750	0.40	0.44	0.42	0.70
Naïve Bayes (1:1)	0.773	0.41	0.46	0.44	0.71
DeepEP	0.826	0.58	0.52	0.55	0.82

### Ablation study

**Fig. 4:**
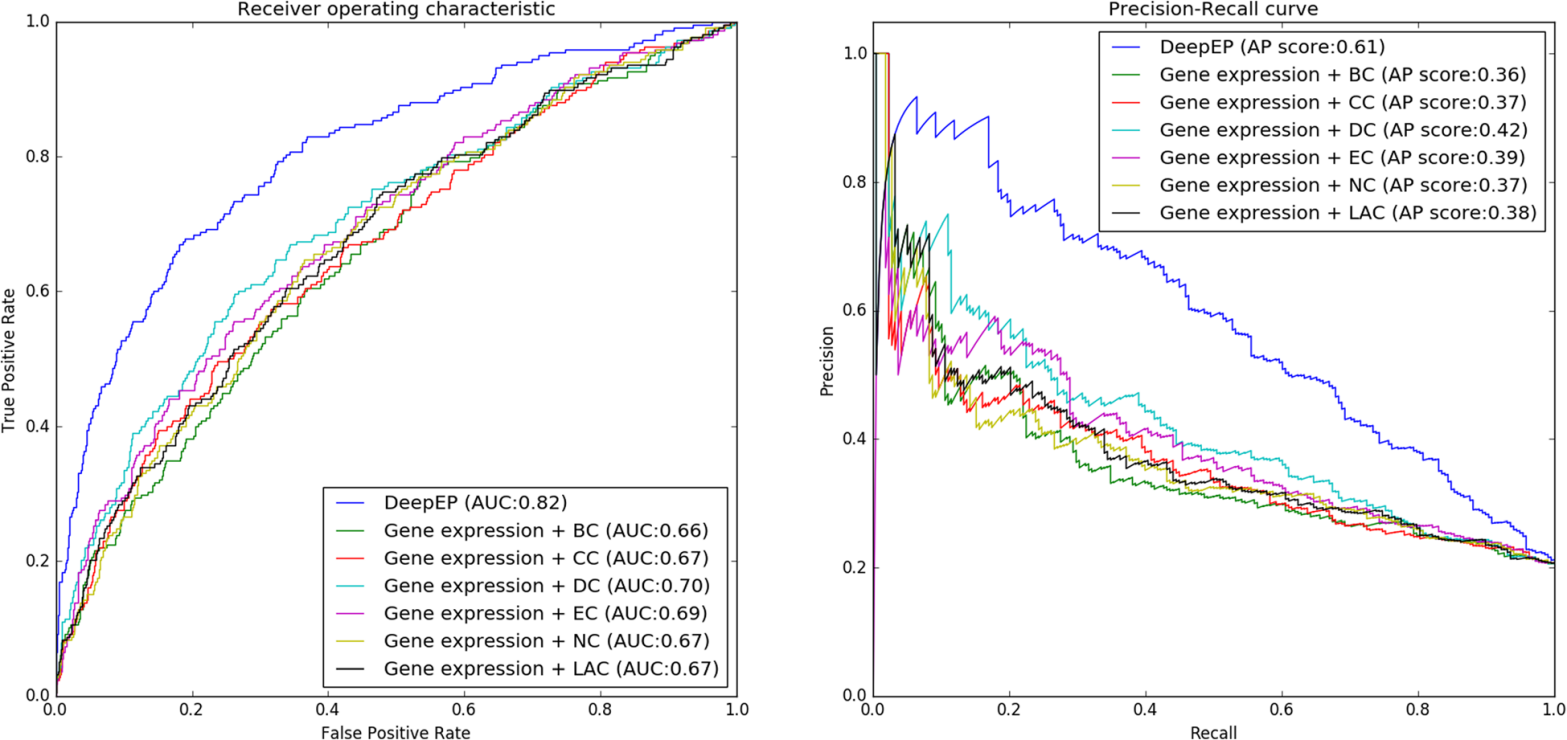
ROC and PR curves of DeepEP and models which use gene expression data combined with different central indexes (DC, CC, EC, BC, NC and LAC)

Our experimental results show that DeepEP improves the performances of identifying essential proteins and outperforms other existing methods. To discover the vital element of DeepEP, we perform experiments by substituting node2vec technique with 6 common used central indexes and the proposed sampling method with different ratios of the positive samples to negative samples in our deep learning framework. In Table 2 we compare the performances obtained by using node2vec technique with the results of traditional central indexes (DC, CC, EC, BC, NC, and LAC). We use a python library called networkx to calculate the six central indexes of PPI network as the network representation of PPI. The rest part of deep learning framework stays the same settings. From Table 2, we can clearly see that node2vec technique is the most effective component and therefore is a crucial element in our deep learning framework. By using node2vec technique, F-measure and AUC of DeepEP are 0.552 and 0.816, respectively, which are better than gene expression data with DC (0.315 and 0.701), CC (0.318 and 0.667), EC (0.348 and 0.690), BC (0.296 and 0.657), NC (0.311 and 0.670), and LAC (0.302 and 0.672). Other metrics of accuracy, precision and recall obtained by node2vec technique are 0.826, 0.584 and 0.524, respectively, which are the highest among all methods. Figure 4 plots the ROC and PR curves of DeepEP and comparing models which use gene expression profiles combined with different central indexes (DC, CC, EC, BC, NC, and LAC). It is evident that DeepEP has the best ROC curve and highest AUC value. Moreover, the AP score of DeepEP is 0.61, which outperforms DC (0.42), CC (0.37), EC (0.39), BC (0.36), NC (0.37), and LAC (0.38). These results indicate that the node2vec technique captures better network features than traditional central indexes. A single central index of PPI network makes use of a single scalar to represent the complex topological features of a protein. Instead, node2vec technique projects a PPI network to a low-dimensional space and generates a dense vector for a protein, and hence it can have richer representation of network topology. In the node2vec technique, vertices are mapped to a low-dimensional space of features which maximizes the likelihood of network neighborhoods of vertices. It makes use of biased random walk technique to efficiently explore diverse neighborhoods and thus the diversity of connectivity patterns in networks are captured, which is the key step to learning richer representations.

**Table 2:** Performances of DeepEP and comparing models (using gene expression profiles combined with different central indexes (DC, CC, EC, BC, NC, and LAC))

Model	Accuracy	Precision	Recall	F-measure	AUC
Gene expression + DC	0.803	0.558	0.220	0.315	0.701
Gene expression + CC	0.782	0.446	0.247	0.318	0.667
Gene expression + EC	0.774	0.429	0.293	0.348	0.690
Gene expression + BC	0.789	0.474	0.215	0.296	0.657
Gene expression + NC	0.779	0.432	0.243	0.311	0.670
Gene expression + LAC	0.796	0.533	0.211	0.302	0.672
Gene expression + node2vec	0.826	0.584	0.524	0.552	0.816

**Fig. 5:**
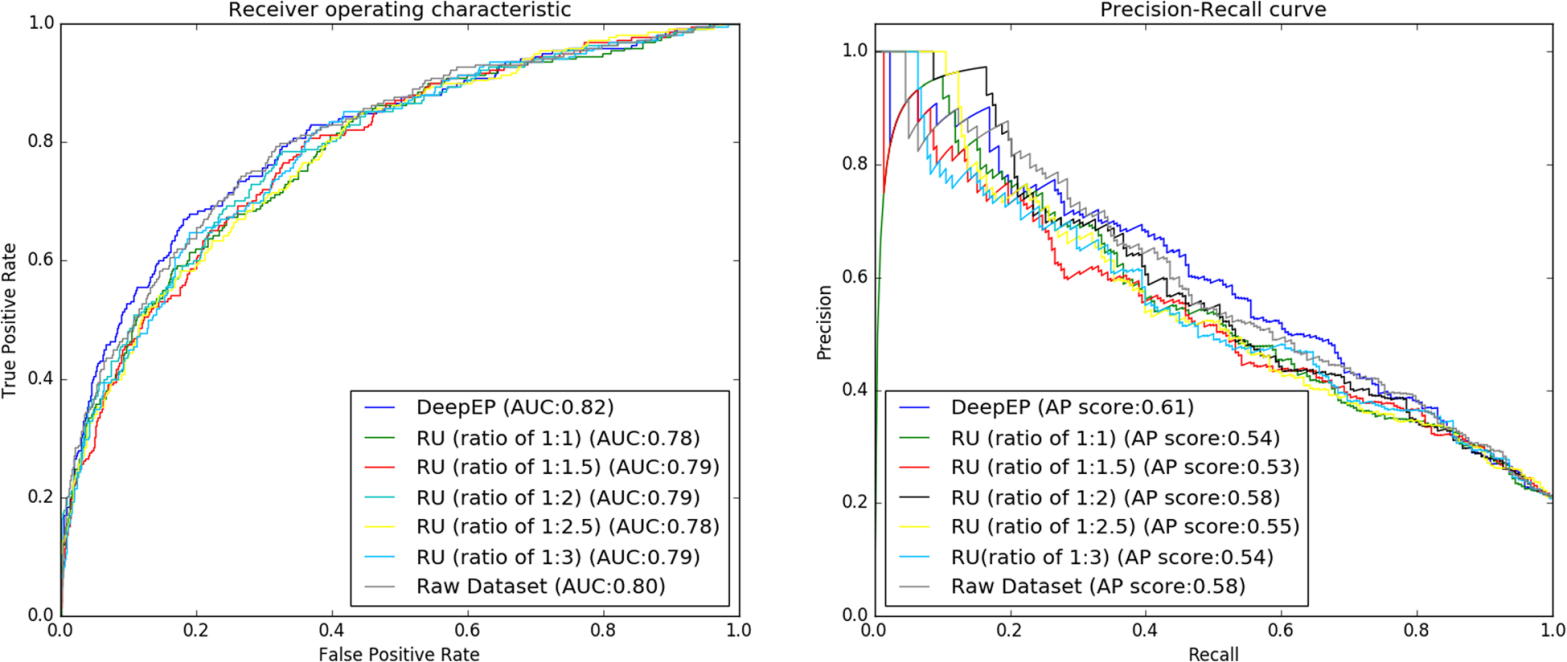
ROC and PR curves of DeepEP, our deep learning framework using different ratios of essential proteins to non-essential proteins (1: 1, 1: 1.5, 1: 2, 1: 2.5 and 1: 3), and using raw dataset. Note: RU refers to random undersampling

We tested the performance of models by using random undersampling technique with different ratios. Random undersampling technique is employed to obtain different datasets which have different ratios of essential proteins to non-essential proteins from raw training dataset. Then different datasets are applied to train different deep learning framework. Specifically, we train our models with different ratios (1:1, 1:1.5, 1:2, 1:2.5 and 1:3) and raw dataset and their performances are given in Table 3. It can be seen that the sampling method is a crucial element in DeepEP. By using the sampling method, F-measure and AUC values obtained by DeepEP are 0.552 and 0.816, respectively, which are better than the ratio of 1:1 (0.508 and 0.783), ratio of 1:1.5 (0.507 and 0.785), ratio of 1:2 (0.510 and 0.791), ratio of 1:2.5 (0.511 and 0.783), ratio of 1:3 (0.482 and 0.788) and using raw dataset (0.463 and 0.803). The ROC and PR curves of comparing methods are shown in Fig. 5. We can see that the ROC curve of DeepEP is slightly higher than those of different ratios. In addition, we can see that the AP score obtained by DeepEP is 0.61, which is obviously higher than 1:1 (0.54), 1:1.5 (0.53), 1:2 (0.58), 1:2.5 (0.55), 1:3 (0.54) and raw dataset (0.58). These two figures also demonstrate that DeepEP works better than random undersampling sampling method with different ratios due to the sampling method. Our analysis shows that two main factors contribute to the better performance of the sampling method. First, we utilize a balanced subset for training in each training epoch, thus our classifier does not bias to any class in each training batch. Second, we make use of all non-essential protein samples in high probability and hence, we do not lose any information of raw dataset.

**Table 3:** Performance of DeepEP and comparing methods (models with different ratios (1:1, 1:1.5, 1:2, 1:2.5 and 1:3) and a model which uses raw dataset for training)

Ratios (Essential VS non-essential)	Accuracy	Precision	Recall	F-measure	AUC
1: 1	0.732	0.408	0.674	0.508	0.783
1: 1.5	0.758	0.437	0.605	0.507	0.785
1: 2	0.784	0.479	0.545	0.510	0.791
1: 2.5	0.796	0.504	0.518	0.511	0.783
1: 3	0.801	0.521	0.449	0.482	0.788
Raw dataset	0.832	0.675	0.353	0.463	0.803
Our method	0.826	0.584	0.524	0.552	0.816

## Conclusions

We propose a new deep learning framework, DeepEP, which is used for identifying essential proteins. DeepEP aims to investigate whether deep learning and sampling methods could achieve notable improvements for identifying essential proteins. The topological features of PPI networks are difficult captured by traditional methods. DeepEP utilizes the node2vec technique to automatically learn complex topological features from PPI network. The node2vec can project the PPI network to low-dimensional space and obtain the representation of proteins with low-dimensional vectors, which allow DeepEP to address the limitations of the traditional methods. In addition, the essential proteins prediction is an imbalanced learning problem; a sampling method is applied in DeepEP to handle this issue. The experimental results obtained by DeepEP show that the proposed approach is able to achieve the state-of-the-art performances that are higher than those obtained by other centrality methods and shallow machine learning-based methods. To understand why DeepEP works well for identifying essential proteins, we conduct studies by substituting node2vec technique with 6 common used central indexes and the proposed sampling method with different ratios. Experimental results show that the dense vectors which are generated by node2vec technique contribute a lot to the improved performance. In addition, the sampling method also helps to improve the performance of deep learning framework.

## Data Availability

The DeepEP source code is available at https://github.com/CSUBioGroup/DeepEP.

## Abbreviations

AUC: Area Under receiver operating characteristic Curve
CNN: Convolutional neural network
PPI: Protein-protein interaction
PR: Precision-recall
RF: Random forest
ROC: Receiver Operating Characteristic
SVM: support vector machine
